# Supplementary figures and images for: Perivascular RELMα-positive synovial macrophages recruit monocytes at the onset of inflammatory arthritis
Source: Front Immunol. 2025 Apr 17;16:1567661. doi: 10.3389/fimmu.2025.1567661 (PMC12043459; doi:10.3389/fimmu.2025.1567661)

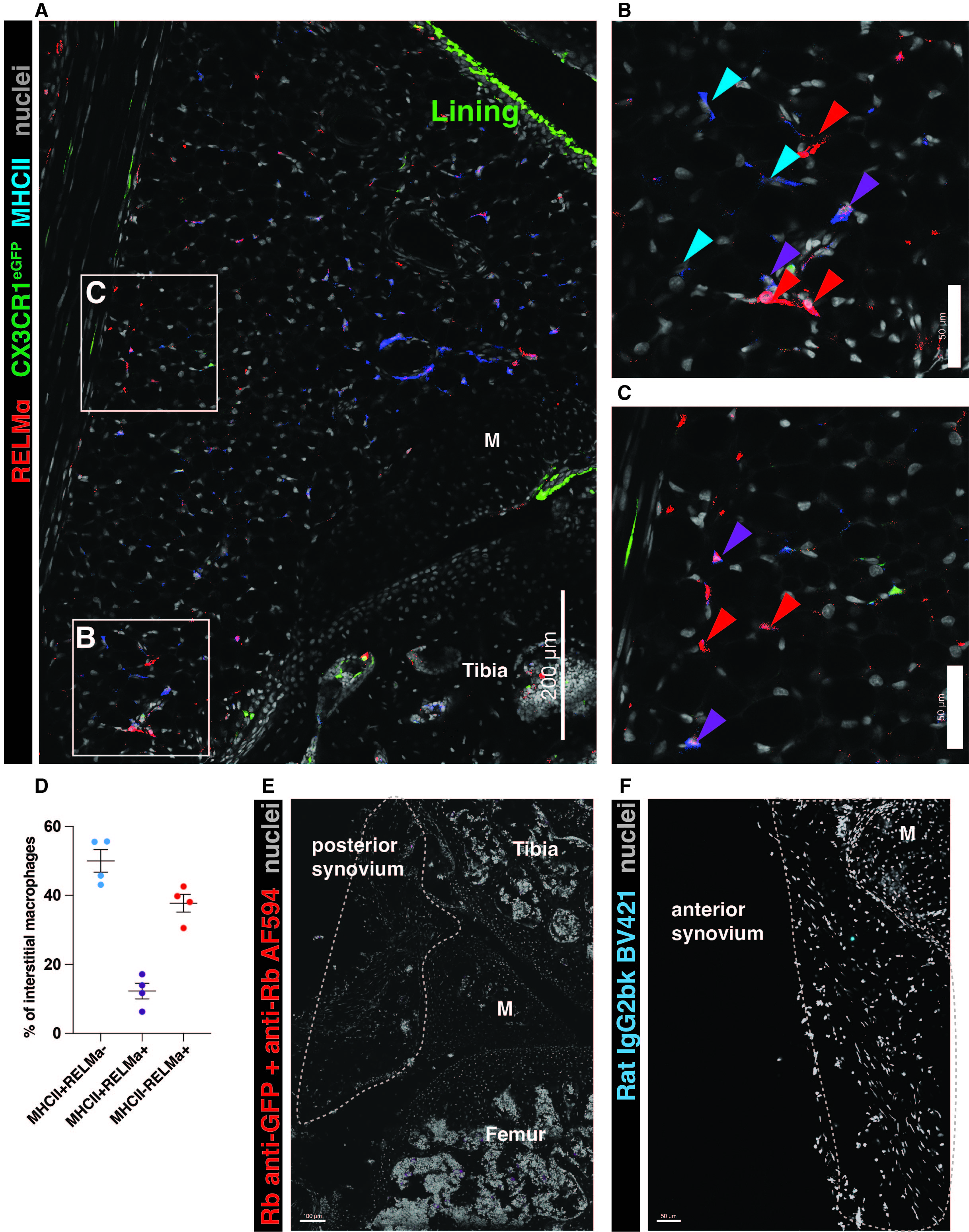

Supplement: Supplementary Figure 1 — MHCII and RELMα colocalise in some, but can mark different subsets of synovial macrophages. (A) A representative image of the naïve synovium, MHCII and RELMα are co-stained and displayed as a pseudochannel with pan-macrophage marker IBA1 to denote macrophages only. The CX3CR1eGFP marks lining macrophages; M=meniscus. (B-C) ROIs highlighted in (A), red arrow indicates RELMα-positive macrophages, the blue arrow marks MHCII-positive macrophages, and purple arrow marks macrophages double-positive for both RELM and MHCII. (D) Quantification of the MHCII+RELM-, MHCII+RELM+, and MHCII-RELM+ subsets as % within synovial interstitial macrophages. Each dot represents an average from positional duplicates in 1 biological replicate (n=4 biological replicates, males). (E-F) Isotype controls for the RELMα (E) and MHCII (F) staining. The control for RELMα staining was done with BL6 wild-type mouse stained with anti-GFP rabbit-derived polyclonal antibody, which was then stained by secondary anti-Rabbit Alexa Fluor 594 antibody. The control for MHCII was done with an isotype control IgG2bĸ BV421. [file Image1.jpeg]

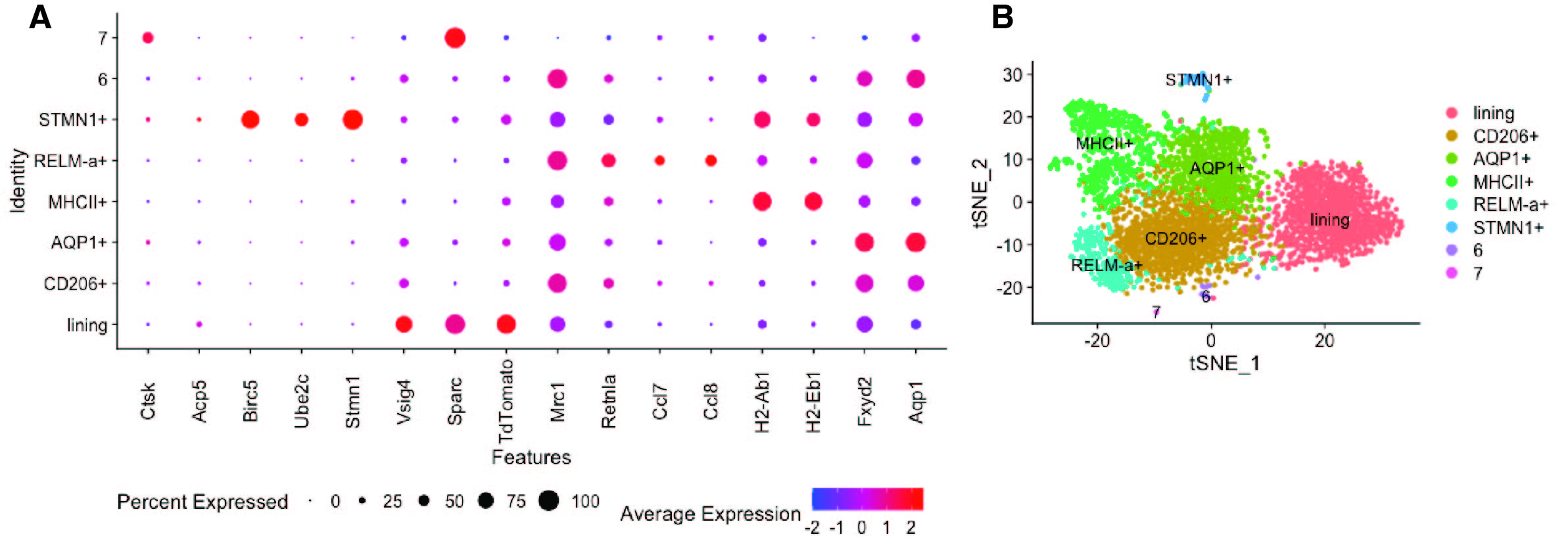

Supplement: Supplementary Figure 2 — Identification of synovial macrophage subsets from a publicly available dataset, from day 1 of STIA (3). (A) Dotplot of markers used for cluster labelling. The selection of key markers was based on the original publication of the dataset.(B) tSNE plot showing the identified populations from STIA day 1. [file Image2.jpeg]

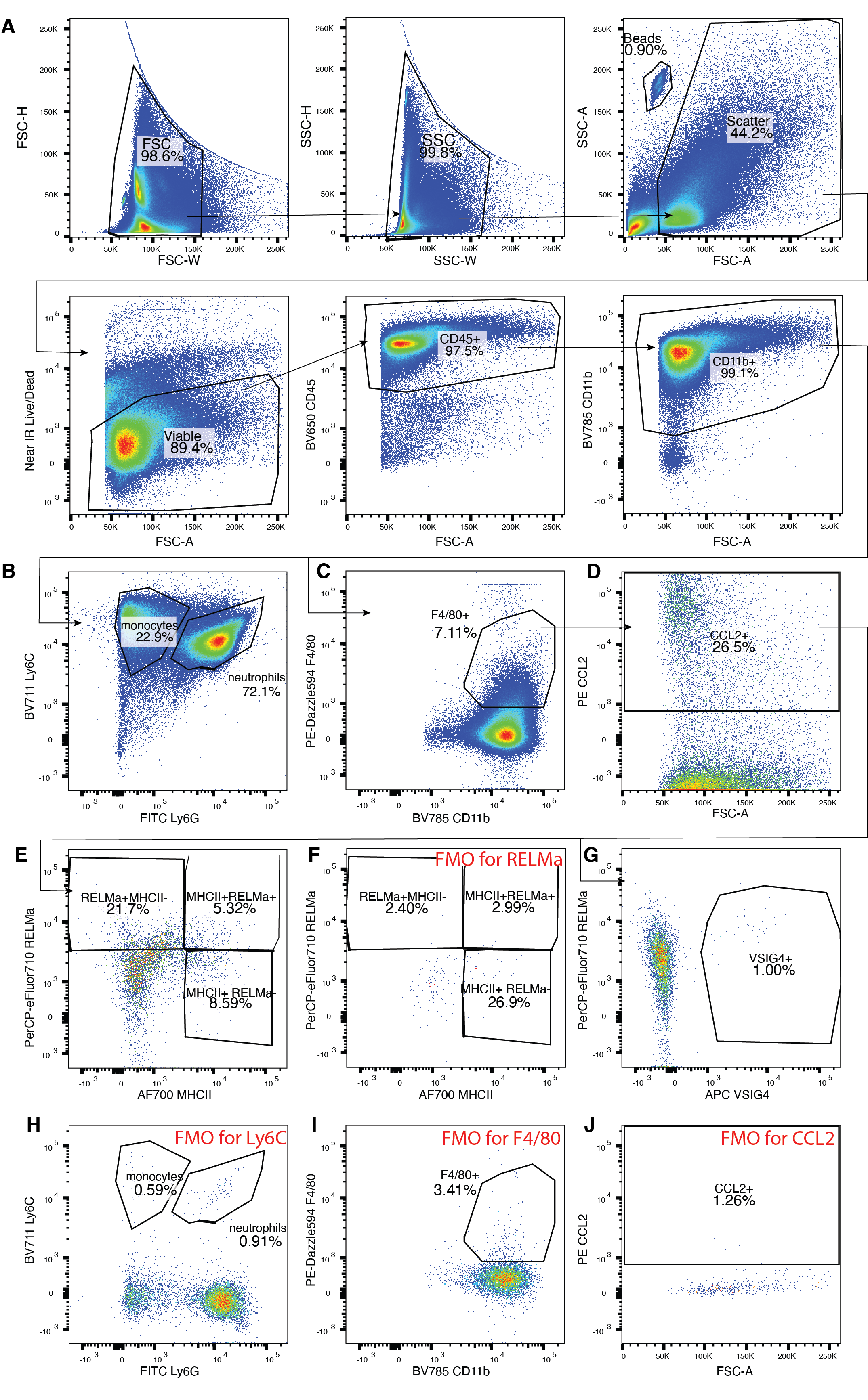

Supplement: Supplementary Figure 3 — Gating strategy to identify CCL2-producing macrophages at day 1 of AIA. (A-D) Identification of the myeloid fraction (A, viable CD45+CD11b+), monocytes (B, viable CD11b+Ly6C+Ly6G-), neutrophils (B, viable CD11b+Ly6C+Ly6G+), macrophages (C, viable CD11b+Ly6C-Ly6G-F4/80+), and CCL2-producing macrophages (D, viable CD11b+Ly6C-Ly6G-F4/80+CCL2+). (E-F) Gating for RELMα+, MHCII+, and RELMα+MHCII+ with FMO control for RELMα (F). (G) Identification of VSIG4+ macrophages. (H-J) FMO controls for Ly6C (H), F4/80 (I), and CCL2 (J) staining. [file Image3.png]
