# Supplementary material for: Perivascular RELMα-positive synovial macrophages recruit monocytes at the onset of inflammatory arthritis
Source: Front Immunol. 2025 Apr 17;16:1567661. doi: 10.3389/fimmu.2025.1567661 (PMC12043459; doi:10.3389/fimmu.2025.1567661)
Supplement: Supplementary file 5 [file Table1.docx]

**Supplementary Table 1: Primary and secondary antibodies used.**

| **Epitope** | **Colour** | **Clone** | | **Manufacturer** | **Cat #** | **Dilution used** |
| --- | --- | --- | --- | --- | --- | --- |
| CCL2 | PE | 2H5 | | Biolegend | 505903 | 1/50 |
| CD11b | BV785 | M1/70 | | Biolegend | 101243 | 1/200 |
| CD31 | BV421 | MEC 13.3 | | BD Biosciences | 562939 | 1/80 |
| CD45 | BV650 | 30-F11 | | Biolegend | 103151 | 1/100 |
| CD68 | Unconjugated | FA-11 | | Biolegend | 137001 | 1/200 |
| F4/80 | PE/Dazzle^TM^ 594 | BM8 | | Invitrogen | 123145 | 1/200 |
| Live/Dead | Near-IR | N/A | | Invitrogen | L10119 | 1/500 |
| Ly6C | BV711 | HK1.4 | | Biolegend | 128012 | 1/100 |
| Ly6G | FITC | 1A8 | | Biolegend | 127605 | 1/100 |
| mCherry | AF594 | 16D7 | | Invitrogen | M11240 | 1/200 |
| MHCII | AF700 | M5/114.15.2 | | Biolegend | 107622 | 1/100 |
| MHCII | BV421 | M5/114.15.2 | | Invitrogen | 404-5321-82 | 1/100 |
| RELM⍺ | PerCP-eF710 | DS8RELM | | Invitrogen | 46-5441-82 | 1/100 |
| RELM⍺ | Unconjugated | Polyclonal | | Abcam | ab39626 | 1/80 |
| RFP | Unconjugated | Polyclonal | | Invitrogen | R10367 | 1/400 |
| VSIG4 | APC | NLA14 | | Invitrogen | 17-5752-82 | 1/100 |
| VSIG4 | Unconjugated | Polyclonal | | Bio-Techne (R&D Systems) | AF4674 | 1/80 |
| **Secondary antibodies** | | | | | | |
| **Colour** | **Species reactivity** | | **Manufacturer** | | **Cat #** | **Dilution used** |
| BV421 | Goat | | Stratech Scientific Ltd | | 705-675-147 | 1/20 |
| AF488 | Rat | | Invitrogen | | A-21208 | 1/400 |
| AF594 | Rabbit | | Invitrogen | | A-21207 | 1/400 |
| AF647 | Rabbit | | Invitrogen | | A32795 | 1/400 |
